# Insights of OPs and PYR cytotoxic potential *Invitro* and genotoxic impact on *PON1* genetic variant among exposed workers in Pakistan

**DOI:** 10.1038/s41598-022-13454-0

**Published:** 2022-06-09

**Authors:** Iffat Imran, Asma Ansari, Saima Saleem, Abid Azhar, Sitwat Zehra

**Affiliations:** The Karachi Institute of Biotechnology and Genetic Engineering (KIBGE), University of Karachi, Karachi, 75270 Pakistan

**Keywords:** Cell biology, Chemical biology, Genetics, Molecular biology, Environmental sciences, Health occupations, Risk factors

## Abstract

Different pesticide chemicals are used to enhance crop yield by protecting from pests. Organophosphate (OPs) and Pyrethroid (PYR) are used in fields of Sanghar, Sindh Pakistan. PON1 an antioxidant enzyme implicated in OPs detoxification may predispose by OPs chronic exposure. This study was conducted to evaluate the toxic potential of active pesticide chemicals at cellular and genetic levels. To examine toxic potential, locally consumed pesticide n = 2 and reference pesticide compounds organophosphate (OPs): Chloropyrifos, Malathion and Pyrethroid (PYR): Cyprmethrin, Cyhalothrin n = 4 were tested against NIH 3T3 cells using MTS assay. Local consumer pesticides demonstrated relevance for half-maximum inhibitory concentration (IC50) 0.00035 mg/mL with selected compound. Malathion IC50 exhibited the highest cytotoxicity among four compounds at 0.0005 mg/mL. On genotoxicity analysis in exposed subjects n = 100 genotypes and alleles n = 200 exhibited significant differences in genotypic and allelic frequencies of pesticide exposed subjects and controls n = 150 (X^2^ = 22.9, *p* = 0.001). Screening of genotypes were performed by PCR- RFLP. Statistical assessment carried out using online software and tools. Results suggested that higher heterozygous genotype A/G (74%) may confer low PON1 metabolic activity towards pesticides in exposed subjects. Findings could be helpful to establish health plans by avoiding toxic chemicals that harming exposed population.

## Introduction

Different pesticide chemicals are used to enhance crop yield by protecting them from insects, weeds and rodents. For this purpose, use of various pesticide chemicals is increasing day by day that are possibly hazardous to human health^1^. Ninety percent of used chemicals in agriculture influence toxic effects on the environment and human health^2^. According to our survey assessment two classes of pesticides organophosphate (OPs) and Pyrethroid (PYR) are mostly used in agricultural fields of Sanghar, Sindh Pakistan. It has been reported that the toxic effects of OPs may inhibit the action of enzyme acetylcholinesterase, which implicates in the hydrolysis of a neurotransmitter the acetylcholine (Ach) that transfers the nerve impulses from the brain to the body. The proficiency of ACh to correspond the response of neuronal systems, affects by accumulation of ACh in the intersynaptic spaces makes cholinergic variation under a complicated mechanism. Accumulation of acetylcholine may lead to termination of conduction between the brain and the body leads to paralysis of an organism^3–5^. There is also evidence for OPs mutagenicity, the European Union has already banned the use of OPs active compounds because of their carcinogenic and mutagenic properties^6–8^. According to previous studies, an association between OPs pesticides chronic exposure and risk for developing various diseases including childhood acute lymphoblastic leukaemia^9^, prostate and lung cancer has been observed^10,11^. Another class of pesticide is PYR extensively used in agriculture and household application. It has also been reported that long term and continuous exposure to PYR may alter the polarization of nerve cell channels and also capable to depolarize membrane^12^. Several epidemiological studies reported that PYR is associated with the risk for childhood acute lymphoblastic leukaemia, childhood brain tumors^13^ and Parkinson`s disease^14^. Continuous and chronic exposure to pesticides may impair the cell detoxification system via inhibition of antioxidant enzymatic activity. In human, the *PON1* gene (7q21.3–22.1) is encoded an antioxidant enzyme Paraoxanase (PON1) plays an important role for the hydrolysis of pesticides. It involves in detoxification of pesticide compounds specifically OPs-Oxon^15^ that is a reactive metabolite of OPs produces neurotoxic effects^16^. The gene *PON1* has been reported is one of the susceptible factors to environmental toxicants including toxic pesticides. Mechanism of detoxification may be altered by a single nucleotide polymorphism, 163 T > A (*rs854560*) results in non- synonymous substitution of methionine (M) to leucine (L) at position 55 (L55M) which demonstrates more sensitive to pesticide toxicity^17^^.^ Upon chronic exposure to pesticide and low hydrolytic activity of PON1 risk for initiation of certain diseases has been indicated by various previous studies including diabetes, neurological disorders, respiratory distress, Alzheimer, dementia and certain cancers^18–22^^**.**^ In current study a survey assessment was conducted among pesticide exposed subjects of Sanghar, Sindh, Pakistan according to recorded facts, dermal absorption of pesticide was found the most common route of entry. Thus a fibroblast cell line NIH/3T3 was chosen for testing of close assessment of selected pesticide toxic effects. The NIH/3T3 cells (originated from the murine fibroblast) has characteristics to grow rapidly, anchorage dependent, contact inhibited and longtime existence in appropriate conditions^23^.This study was conducted to recognize and assess the toxic effects of pesticide chemicals could have an impact at cellular (dosage dependent) and genetic (dosage in-dependent) levels.

## Results

### Cytotoxic potential of local consumer pesticide solution

**Figure 1:**
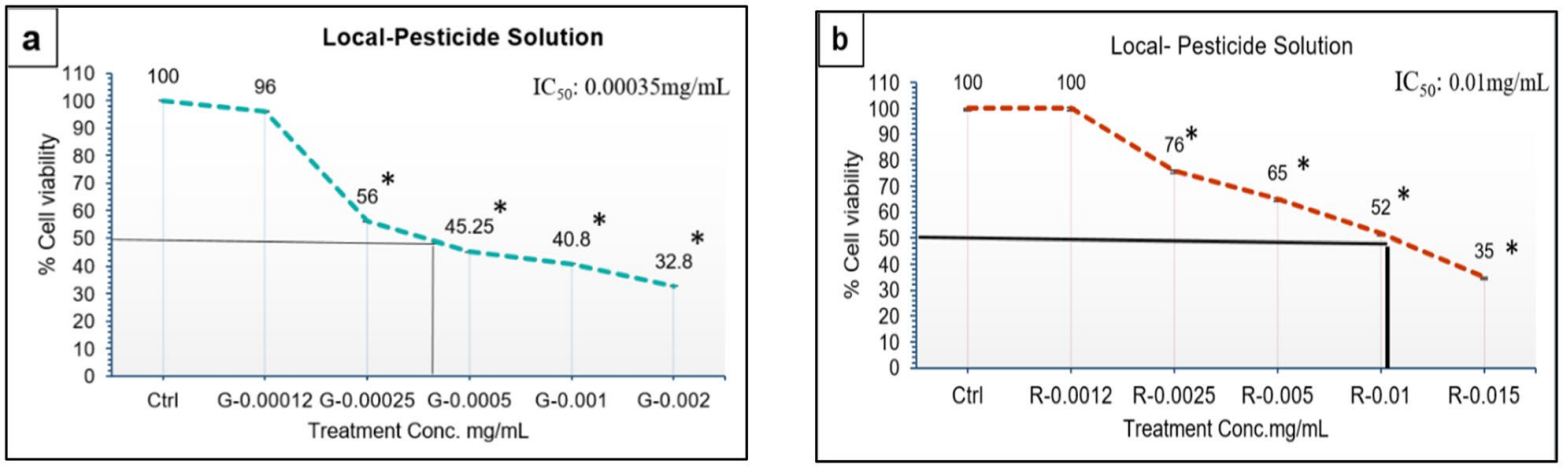
Mean cell viability of NIH 3T3 cells after 24 h exposure at different specific concentrations of Local consumer pesticide solution in mg/mL. (**a)** Green solution (local pesticide) specific dose treatments. (**b)** Red solution (local pesticide) specific dose treatments . ANOVA and LSD post-hoc analyses were used to compare means of the treated cells with untreated control cells. Data were presented as Standard Error of Mean (Mean ± SEM) of triplicate determinations. The mean difference is significant when *p < 0.001. Ctrl: control, G: green local pesticide solution; R: red local pesticide solution; IC50: maximum half inhibitory concentration (mg/mL).

Two local pesticide solutions “green” and “red” colored were tested for their toxic potential. After 24 h of exposure to green pesticide solution a significant difference at dose 0.00035 mg/mL on cell viability was observed (Fig. 1a). After treatment with red pesticide solution it was observed that significant effect at dose 0.01 mg/mL was exhibited on cell viability when compared with control cells (Fig. 1b).(Green pesticide solution: pack stock concentration 0.01 mg/mL, red pesticide solution: pack stock concentration 0.02 mg/ mL).

### Cytotoxic potential of reference pesticides

**Figure 2:**
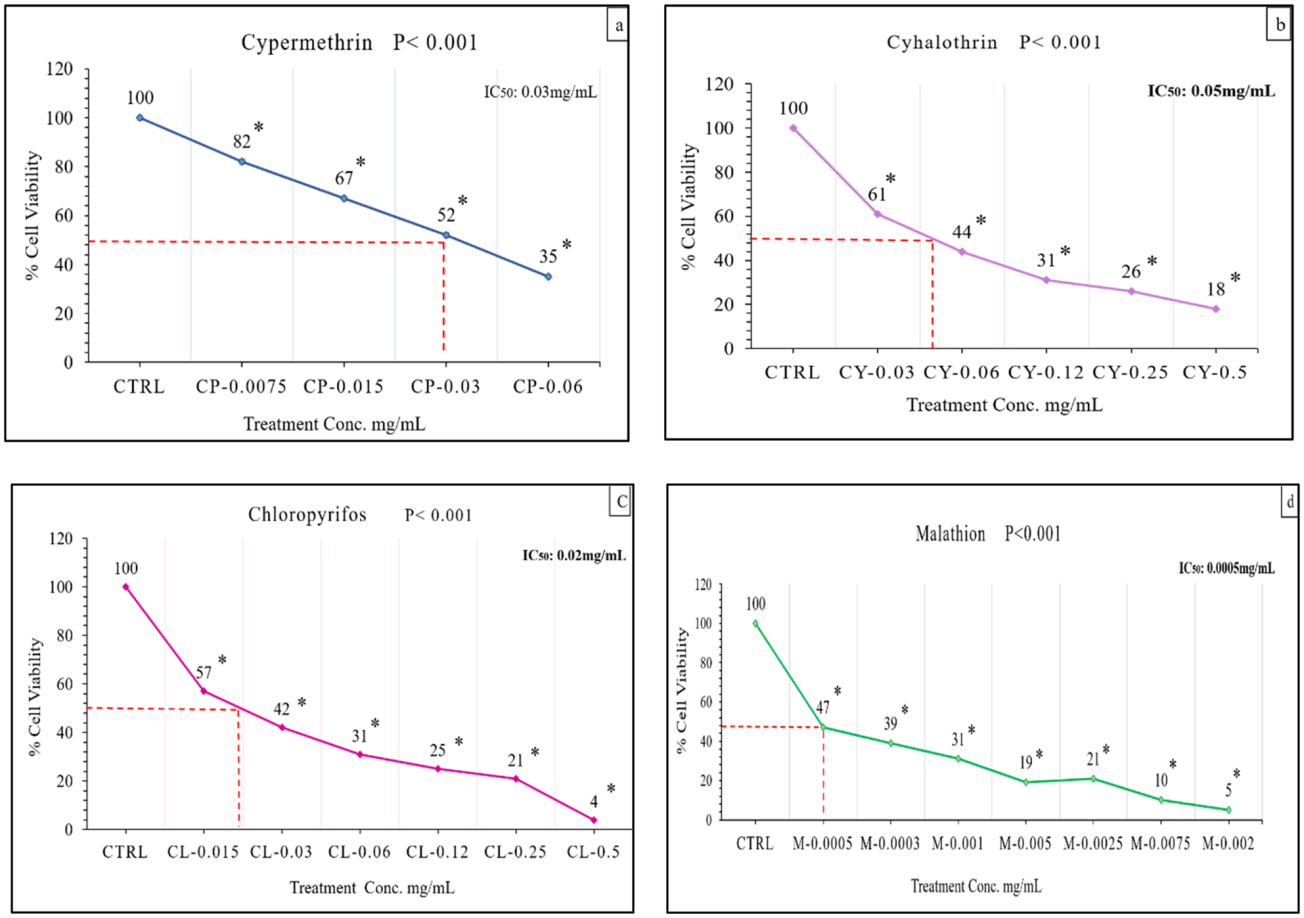
Mean cell viability of NIH 3T3 cells after 24 h on exposure to different specific concentrations of PYR pesticide in mg/mL. **(a)** Cypermethrin (PYR) (**b)** Cyhalothrin (PYR) **(c)** Chloropyrifos (OPs) (**d)** Malathion (OPs). ANOVA and LSD post-hoc analyses were used to compare means of the treated cells with untreated control cells. Data were presented as Standard Error of Mean (Mean ± SEM) of triplicate determinations. The mean difference is significant when *p < 0.001. n= 4, CTRL: Control, CP: Cypermethrin, Cy: Cyhalothrin; Cl: Chloropyrifos, M: Malathion; IC50: maximum half inhibitory concentration (mg/mL).

MTS assay was performed to examine the influence of PYR and OPs pesticide reference chemicals on NIH/3T3 cells at dose dependent manner. Regarding PYR pesticides after 24 h of exposure to specific treatments of Cypermethrin, it was observed that at dose 0.03 mg/mL significant inhibition was observed on cell viability (Fig. 2a). On exposure to Cyhalothrin against NIH/3T3 cells at gradient concentrations it was demonstrated that a significant difference in half cell viability at dose 0.05 mg/mL was calculated when compared with control cells (Fig. 2b). Different concentrations of OPs pesticides Chloropyrifos was determined for its toxic potential a significant decrease in cell viability after 24 h was observed at dose 0.02 mg/mL (Fig. 2c). whereas OPs Malathion was exhibited half reduction of cells at dose 0.0005 mg/mL (toxic potential: Malathion > Chloropyrifos > Cypermethrin > Cyhalothrin) (Fig. 2d).

### Association analysis in study subjects

Association of *PON1* genetic (*rs854560*) variant with OPs toxicity defined in (Table 1). Genotypic frequency distribution between exposed and control subjects was estimated by Chi-square (*p* < 0.05), was done according to Hardy- Weingberg equiliberium (HWE) law. In genotypic distribution, the higher occurrence of T/T and A/A genotypes were demonstrated in controls whereas the T/A genotype was indicated higher among exposed (74%) as compared to controls (43%) the results negates HWE (*p* < 0.05). Allelic distributions revealed that mutant allele “A” was higher in frequency in the exposed subjects (39%). Our study suggested that in *PON1* genetic variant (*rs854560*) mutant allele “A” showed a significant resistance against OPs toxicity in exposed subjects (OR = 0.47, 95% CI: 0.25– 0.87, *p* = 0.01).

**Table 1:** Genotypic and allelic freuencies of *PON1* rs854560 in study subjects with OPs and PYR pesticide toxicity.

SNP	Genotype n = 250	Exposed n = 100	Control n = 150	Chi-square	p-value
***PON1*** rs854560	TT	24 (24%)	77 (51%)	22.9	0.001***
	TA	74 (74%)	65 (43%)		
	AA	2 (2%)	8 (5%)		
	**Alleles**	n = 200	n = 300	**Odds ratio (95%CI)**	**p-value**
	T	122 (61%)	146 (48%)	0.473 (0.25- 0.87)	0.016
	A	78 (39%)	54 (18%)		

Significance *p* < 0.05.

## Discussion

Pesticide toxicity is countered through various detoxification mechanisms in human. These mechanisms comprise of oxidases, hydrolases, and transferases. The actions of involved enzymes is influenced by genetic variations^16^. In the current study pesticide compounds were selected through a survey questionnaire according to this Chloropyrifos, Malathion (OPs), Cypermethrin and Cyhalothrin (PYR) are frequently applied in the fields of Sanghar, Sindh, Pakistan. Local consuming pesticide solutions (commercially branded) were also collected from the applying fields. To check the toxic potential of certain reference compounds at dose-dependent manner a cell viability assay (MTS) was employed. On the other hand toxicogenic association with *PON1* (*rs854560*) variant and tested pesticide compounds, genotypes of local exposed subjects were investigated for genetic variations. As mentioned above PON1, in particular, involved in the hydrolysis of a variety of OPs but predominantly acts on their metabolites like Oxon. Upon pesticide continuous exposure a change in expression and efficiency of PON1 has been reported in various studies^24,25^. PON1 provides several physiological roles in the metabolism of OPs and causes oxidative stress^26^. Inhibition of PON1 due to environmental factors affects quantitative or qualitative synthesis of PON1 may cause serious health concerns in exposed individuals^27–29^. Some endogenous and exogenous elements are responsible to obstruct pesticide metabolism. Due to single nucleotide polymorphism (SNP) individuals either sensitive or resistant to pesticide exposure^30^. Sözmen et al. demonstrated an inhibition in PON1 activity in OPs exposed workers, that inhibition was independent of the pesticide type and amount of the OPs. It was also indicated that *PON1* polymorphisms are crucial risk factors in vulnerability to OPs toxicity and may be used as a marker of chronic exposure to pesticides in farmers^31^.Thus the effect of OPs response on *PON1* (*rs854560*) variant was investigated in this study as an anticipated susceptible factor in the exposed group. Exposed individuals had more than 2 years of employment period with selected pesticide were recruited in this study. As results suggested that among four reference compounds Malathion (OPs) showed close IC50 dose relevant with locally collected pesticide solution. Therefore the study suggested that OPs may be associated with *PON1* *(rs854560*) polymorphism. It was obvious that *PON1* (*rs854560*) genotypic frequencies distribution showed a strong significant association with *PON1* (rs854560) variant and OPs pesticide exposure in the study population of Sanghar, Sindh, Pakistan. In exposed subjects, higher heterozygous genotype A/G (74%) may confer low PON1 metabolic activity towards pesticide metabolism in exposed subjects as compared to wild type genotype T/T (51%) in controls may have higher risk for pesticide toxicity.

Results of current study are consistent with a recent study, conducted between two different populations (Cameron: Figuil, Njobe and Sa'a and Pakistan: Depalpur and Multan) a strong significant difference was observed in *PON1* (*rs854560*) polymorphism in both OPs exposed to agriculture populations (Pakistan: X^2^ = 126.102, p = 0.000) and (Cameron: X^2^ = 32.200, *p* = 0.000)^16^. Our previous study also indicated pesticide-induced polymorphisms in phase1 and phase II pesticide metabolizing genes in subjects of Sanghar, Sindh OPs exposed regions^32^. Numerous studies in Pakistan showed a possible association with health-related conditions in exposed individuals. A cross- sectional study determined an association of pesticides exposure with the disruptive function of lungs in rural Sindh, Pakistan^33^. Intensification or reduction in the levels of the neurotransmitter and exert oxidative damage to neuronal cells has been also been reported in a previous study^34^. Another study demonstrated higher DNA damage in the exposed group as compared to the non-exposed group in Bahawalpur District, Punjab Pakistan^35^. To the best of our knowledge, no prior epidemiologic study has examined active ingredients used in the locally consumed pesticide solution. In vitro toxicity testing may evaluate visible effects at dose dependent manner which may help to assess the invisible effects (dosage independent) *in-vivo*. According to previous in vivo studies specific dose amounts may induce different cellular responses in the pesticide exposed animal models. It has been observed that when rats were exposed to a threshold level (500 mg/kg) delayed neurotoxicity was observed^36^. Other study reported that after 30 min of pesticide exposure (42.5 mg/kg) neurobehavioral changes were observed in whister rats^37^. In another study decreased Acetyle cholinesterase (AChE) was determined immediately after pesticide dose administration (10 mg/kg oral) in rats. Moreover in another study at a similar dose (i.e. 10 mg/kg) DNA fragmentation and apoptotic cell death were also observed in pesticide exposed rats^38,39^. All these previous studies supported modifications at different cellular levels which might have a deleterious impact on health condition.

A significant difference was observed in genotypes between both study groups estimated by Chi- square statistics Table 1. The higher frequency of the A/G genotype of the PON1 variant showed a higher ratio in the exposed group (74%) as compared to the control group (43%). Findings of the study may be supportive for the prevention and to mitigate the associated health risks linked to pesticide chronic exposure in exposed individuals. Heavy usage of pesticide might be obtain higher crop yields further, for sales promotions farmers are encouraged from pesticide companies for heavy application of pesticides^40^. A consistent biological monitoring, estimation and recorded procedures should be executed among occupationally exposed individuals for their risk assessment. At the molecular level dearth of data availability in rural areas of Pakistan is the major hindrance to establishing clear environmental health related-issues. There are limitations in the study; limited sample size, lack of data availability at the molecular level in the selected regions, other environmental factors like arsenic, lead etc. exposures were not focused and ethnic differentiation were also not considered these were the possible bias of the study.

## Conclusions

Among four selected pesticide compounds, *PON1* (*rs854560*) exhibited an anticipated association with Malathion (OPs) toxicity. PON1 enzyme detoxifies OPs metabolites, a single nucleotide polymorphism in the coding region of *PON1,* which may alter the activity and concentration of PON1 may cause serious health disorders in exposed individuals. The findings will be helpful to identify toxic environmental agents, their prevention and also to mitigate the associated health risk linked to pesticide chronic exposure in exposed individuals. Further pharmacogenetic studies might be conducted to elucidate the role of *PON1* (*rs854560*) variant in the mechanism of pesticide detoxification.

## Materials and methods

### Ethical approval and sample collection

This study is an extended part of previously published research^32^. The study was conducted in the month of August 2019 in Shahpur Chakara, District Sanghar, Sindh Pakistan. Helsinki Declaration was followed, the Institutional Ethical Committee of Karachi Institute of Biotechnology and Genetic Engineering (KIBGE), University of Karachi allowed this research (Ref: KIBGE/ICE/114/15/09/2017). The selection of participants was completed by following inclusion and exclusion criteria. It was a case–control study comprised of n = 250 study participants, divided into two study groups exposed n = 100 involved in mixing, treating, stocking, crop plucking and spraying activities in agricultural fields while controls n = 150 comprised of non-occupational subjects Supplementary Table 1. For donor participation, informed consent was taken from all study participants.

### Chemicals and treatments

Cytotoxic potential of pesticide compounds was analysed [**Pyrethroid:** Cypermethrin (CAS No. 52315–7–8), Cyhalothrin (CAS No. 68085–85-8) and **Organo-phosphate:** Chloropyrifos (CAS No. 5598–13-0), Malathion (CAS No.1190–28-9)] were purchased from Sigma Aldrich (St. Louis, USA). Local consumer pesticide solutions “red Karate® (Cypermethrin, pack stock concentration 0.02 mg/ mL) and green” Fyfanon® (Malathion, pack stock concentration 0.01 mg/mL) were collected from sprayer`s container of Sanghar, Sindh. As a solvent DMSO, 99% from SERVA was used to prepare stock solutions. Stock solutions were directly diluted in DMEM from Sigma Aldrich. Methylthiazolyldiphenyl-tetrazolium bromide (MTS ™) dye was purchased from Thermo Scientific, USA. For cytotoxicity analysis of pesticides in this study, compounds were selected on the basis of survey assessment including Lorsban®, Nurella®, Karate®, Soligor®, Agarin®, Hero®, Nufos®, Syngenta®, Perfect killer® and Fyfanon® Supplementary Table 2.

### Cell viability assay

NIH/3T3 (ATCC® CRL-1658™) Fibroblast cells were used, the cytotoxic potential of pesticides was evaluated using MTS cell viability assay^41^. In DMEM cell growth medium 1 × 10^5^ cells /mL were seeded then a micro-culture plate was inoculated with 100µL/well, incubated for 24 h at 37 °C in 5% CO_2_ humidified incubator (ESCO CO_2_). After 24 h, cells were treated with different concentrations of selected pesticide chemicals. All pesticide compounds and solutions were tested in the triplicate individual experiments. Culture plates were placed for 24 h at 37 °C for incubation. The medium was removed after 24 h and then 20µL of MTS solution was added to cell culture, incubated for 4 h absorbance of viable cells was recorded at 490 nm by ELISA reader (Bio Base EL-10A®). At each specific concentration (IC70, IC50 and IC30) morphological changes were examined under the phase contrast microscope (LEICA DM-IRB) Supplementary Fig. 1.

### DNA extraction and detection of SNP

For extraction of DNA from the whole blood of study subjects Phenol–chloroform, method was performed with few modifications from the previously described method^42^. *PON1* (rs854560) region was amplified using a set of primers chosen from previously reported study^43^. F (forward): 5`TCTGGCAGAAACTGGCTCTGAAGCC3` and R (reverse): 5`CTTAAACTGCCAGTCCTAGAAAACG3` purchased from Eurofins Genomics®, USA. After amplification of a product of 130 bp was obtained (Supplementary Fig. 2a.). The flow of optimized PCR conditions and reagents was the same with slight modification in the annealing temperature of 52 °C described in previous study^32^. For detection of single nucleotide polymorphism in *PON1* (*rs854560*) region, restriction enzyme *NCO1* was selected from NEB cutter V2.0. The amplified product was digested followed by manufacturer`s protocol (ThermoFisher Scientific, USA). Digested products were visualized on 2% agarose gel under a gel documentation system (FastGene® FAS V, Germany) (Supplementary Fig. 2b).

### Statistical analysis

For the estimation of the observed data statistical package for social sciences (SPSS) version 20.0 was used. One-way analysis of variance (ANOVA) statistics was particularly used to compare percent cell viability values at each specific concentration of seven pesticides. For least significant differences (LSD) in the significant percent cell viability values post hoc was used. To determine the differences in genotypic frequencies between pesticide exposed and controls Pearson chi-square (X^2^) test was used considering three-by-three contingency. Allelic association with genetic susceptibility and pesticide exposure was estimated using odds ratio (OR) considering two-by- two contingency using online tool Medcalc®. Results were considered significant when p < 0.05*, p < 0.01**, p < 0.001***.

### Ethics declarations

#### Approval for human experiments

This study was approved and reviewed by the institutional ethical committee (Institutional Review Board (IRB)) Ref: KIBGE/ICE/114/15/09/2017) that approved the experiments including relevant details. Written informed consent was taken prior to blood collection from all study subjects. Further, a designed survey questionnaire was filled from exposed subjects regarding occupational information, pesticide brand names and immediate medical conditions after pesticide spray etc. All experiments were performed in accordance with relevant guidelines and regulations.

## Supplementary Information

Supplementary Information 1.

Supplementary Information 2.

Supplementary Information 3.

Supplementary Information 4.

Supplementary Information 5.

## Data Availability

This research work is an extended part of the previously published work can be accessed (Imran et al. 2021). https://doi.org/10.1007/s11756-021-00698-w.
