# Supplementary material for: Insights of OPs and PYR cytotoxic potential Invitro and genotoxic impact on PON1 genetic variant among exposed workers in Pakistan
Source: Sci Rep. 2022 Jun 9;12:9498. doi: 10.1038/s41598-022-13454-0 (PMC9184543; doi:10.1038/s41598-022-13454-0)
Supplement: Supplementary file 1 — Supplementary Information 1. [file 41598_2022_13454_MOESM1_ESM.docx]

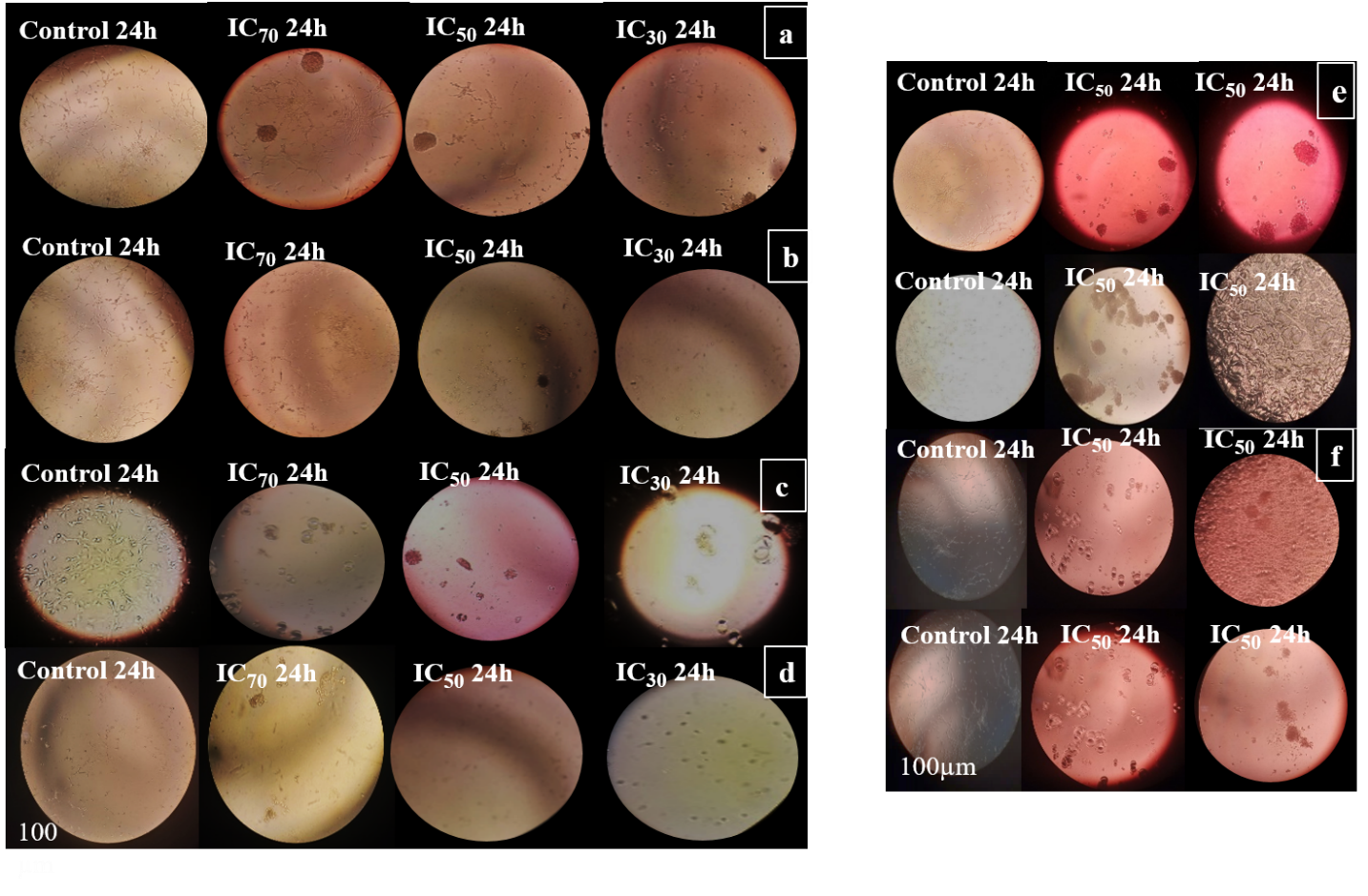


**Figure 1:** Morphological analysis at specific dose exposures of IC70, IC50 and IC30 after 24 hours incubation, compared with control cells **[a]** exposure to Cypermethrin (PYR) **[b]** exposure to Cyhalothrin (PYR) **[c]** exposure to Malathion (OPs) **[d]** exposure to Chloropyrifos (OPs).
