# Supplementary material for: Insights of OPs and PYR cytotoxic potential Invitro and genotoxic impact on PON1 genetic variant among exposed workers in Pakistan
Source: Sci Rep. 2022 Jun 9;12:9498. doi: 10.1038/s41598-022-13454-0 (PMC9184543; doi:10.1038/s41598-022-13454-0)
Supplement: Supplementary file 2 — Supplementary Information 2. [file 41598_2022_13454_MOESM2_ESM.docx]

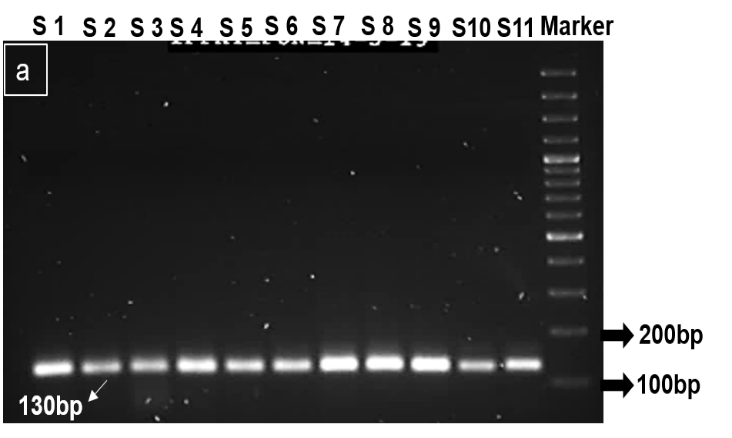


**Figure 2a.** Amplified and digested products *PON1* rs854560 (130bp), Lane 1- 11 amplified products, Lane marker 100bp DNA ladder
