# Supplementary figures and images for: Insights of OPs and PYR cytotoxic potential Invitro and genotoxic impact on PON1 genetic variant among exposed workers in Pakistan
Source: Sci Rep. 2022 Jun 9;12:9498. doi: 10.1038/s41598-022-13454-0 (PMC9184543; doi:10.1038/s41598-022-13454-0)

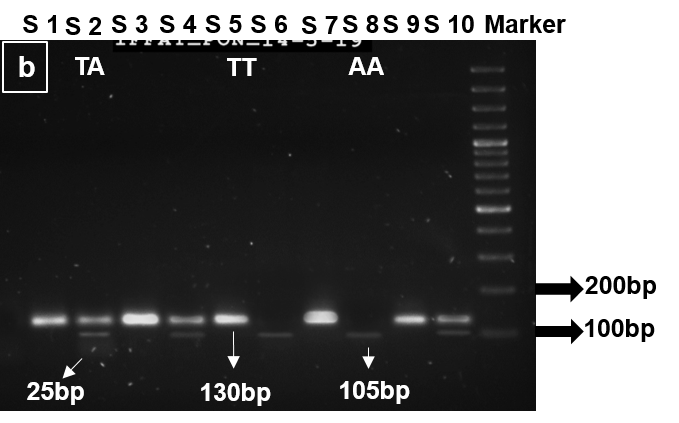


**Figure b:** Digested products *PON1* rs854560, TT: wild type (130bp), TA: heterozygous (130, 105 & 25bp) and AA: mutant (105 & 25bp)

Supplement: Supplementary file 3 — Supplementary Information 3. [file 41598_2022_13454_MOESM3_ESM.docx]
