# Supplementary material for: Insights of OPs and PYR cytotoxic potential Invitro and genotoxic impact on PON1 genetic variant among exposed workers in Pakistan
Source: Sci Rep. 2022 Jun 9;12:9498. doi: 10.1038/s41598-022-13454-0 (PMC9184543; doi:10.1038/s41598-022-13454-0)
Supplement: Supplementary file 4 — Supplementary Information 4. [file 41598_2022_13454_MOESM4_ESM.docx]

| **Characteristics** | **Mean± SD** |
| --- | --- |
| Age, years | 33.9 ±10.9 |
| Weight, kg | 61.5 ± 10.4 |
| Daily exposure, hours | 2.36 ±1.13 |
| Length of employment | 12.64 ± 6.51 |
| **Exposed subjects** | **Percentage** |
| Gender |  |
| Male | 84 |
| Female | 16 |
| Education |  |
| Literate | 59 |
| Illiterate | 41 |
| Follow instructions | 54 |
| Protective measures | 7 |
| Main route of exposures |  |
| Skin | 51 |
| Nasal | 22 |
| Immediate effects | 89 |

**Table 1:** Survey based knowledge regarding exposed subject`s characteristics, occupation, handling practices and health effects
