# Supplementary material for: Insights of OPs and PYR cytotoxic potential Invitro and genotoxic impact on PON1 genetic variant among exposed workers in Pakistan
Source: Sci Rep. 2022 Jun 9;12:9498. doi: 10.1038/s41598-022-13454-0 (PMC9184543; doi:10.1038/s41598-022-13454-0)
Supplement: Supplementary file 5 — Supplementary Information 5. [file 41598_2022_13454_MOESM5_ESM.docx]

**Table 2:** Comparision of tested pesticides (IC50) with commercialy available pesticides (stock conc.) used by Sanghar, Sindh farmers

| **S.No.** | **Name brand** | **Active compound** | **Pack stock concentration** | **Present study IC_50_** |
| --- | --- | --- | --- | --- |
| 1 | Karate-2^®^ | Cypermethrin | 0.02mg/mL | 0.03 mg/mL |
| 2 | Karate^®^ | Cyhalothrin | 0.025 mg/mL | 0.015 mg/mL |
| 3 | Karate^®^ | Cyhalothrin | 0.4 mg/mL | 0.015 mg/mL |
| 4 | Atrazine^®^ | Cyhalothrin | 0.303 mg/mL | 0.2 mg/mL |
| 5 | Agarin^®^ | Cypermethrin | 0.266 mg/mL | 0.25 mg/mL |
| 6 | Syngenta^®^ | Cyhalothrin | 0.4 mg/mL | 0.5mg/mL |
| 7 | Soligor^®^ | Cypermethrin | 0.02 mg/mL | 0.03 mg/mL |
| 8 | Endigo^®^ | Cyhalothrin | 0.05 mg/mL | < 0.06 mg/mL |
| 9 | Hero^®^ | Cypermethrin | 0.102 mg/mL | 0.1 mg/mL |
| 10 | Nufos^®^ | Chloropyrifos | 0.022 mg/mL | **Less toxic** |
| 11 | Lorsban^®^ | Chloropyrifos | 0.018 mg/mL | **Less toxic** |
| ,12 | Karate^®^ | Cyhalothrin | 0.043 mg/mL | 0.03 mg/mL |
| 13 | Hoiser^®^ | Cypermethrin | 0.032 mg/mL | 0.03 mg/mL |
| 14 | Perfect killer^®^ | Chloropyrifos | 0.05 mg/mL | **Less toxic** |
| 15 | Solo love^®^ | Cypermethrin | 0.01 mg/mL | 0.03 mg/mL |
| 16 | Fyfanon^®^ | Malathion | 0.011 mg/mL | 0.0003 mg/mL |

Grey font: Relevance with local pesticide solution (IC50) and commercially available pesticides (stock concentration)
